# Practical Points

**Published:** 1892-07-09

**Authors:** 


### PRACTICAL POINTS.

Questions are invited for this column on any point in the administration of hospitals and asylums which may prove of difficulty or interest to our readers. All communications should be marked "Practical Points," addressed to the Editor of The Hospital, 140, Strand, W.C.

*Convalescent Home Construction.*—"Secretary" writes:—The latest and most complete plans are, I believe, those of the "Fletcher Convalescent Home of the Norfolk and Norwich Hospital," now being built at Cromer, designed by Mr. Edward Boardman, F.R.I.B.A., of Queen Street, Norwich, the architect of the N. and N. Hospital.

*Model Subscription Boxes.*—"*Hon. Secretary*" *is most anxious to procure the best design for hospital subscription boxes. If any reader will supply descriptive account, with design, and state the price, we shall be glad to publish it.*

